# Acute Methemoglobinemia Due to Crop-Flowering Stimulant (Nitrobenzene) Poisoning: A Case Report

**DOI:** 10.7759/cureus.47766

**Published:** 2023-10-26

**Authors:** Yogesh S, Hariharan Seshadri, Umadevi TB, Seethalakshmi N, Navvin S

**Affiliations:** 1 Institute of Internal Medicine, Madras Medical College and Rajiv Gandhi Government General Hospital, Chennai, IND

**Keywords:** methemoglobin, nitrobenzene, nitrobenzene poisoning, agrochemicals, toxicology, toxicology and poisoning, saturation gap, methylene blue treatment, peripheral cyanosis, self poisoning

## Abstract

Nitrobenzene poisoning is an uncommon but serious form of intoxication. Nitrobenzene is used in dyes, paints, lubricating oils, and crop-flowering stimulants. Ingestion produces acute methemoglobinemia and cyanosis, which fails to produce improvement in high-flow oxygen therapy. We present here a case of a 25-year-old male presenting with diffuse headache, fatigue, and cyanosis after attempting suicide by consumption of 15 mL of 20% nitrobenzene. Oxygen saturation (SpO_2_) was 85% on room air and was not improving on oxygen therapy. Serum methemoglobin level was 22% of hemoglobin. The patient was treated with IV methylene blue and oral ascorbic acid along with supportive management. He attained recovery by day three and was subsequently discharged. Acute methemoglobinemia following nitrobenzene poisoning is of grave concern and demands timely identification and diligent management with methylene blue and ascorbic acid alongside supportive measures.

## Introduction

Poisoning is one of the leading causes of death in India. It accounts for a significant proportion of medical emergencies. Pesticides are the major cause of poisoning in adults, with organophosphorus compounds being the most common. Intentional poisoning accounts for the vast majority of cases (more in adult males) and is associated with an underlying primary psychiatric illness, financial setbacks, social and family issues, or an underlying psychiatric illness. Accidental poisoning is more common in children and the poisons consumed are household products or those acquired through environmental exposure. Acute poisoning leads to mortality and severe morbidity and requires intensive medical attention [1]. Nitrobenzene is an oxidizing nitrite compound used in dyes, paint solvents, lubricants, and plant growth stimulants. Acute poisoning with nitrobenzene presents with methemoglobinemia, which is excessive accumulation of methemoglobin (MetHb) in the blood. Between 2020 and 2023, only five cases of nitrobenzene poisoning have been reported at our set-up, Rajiv Gandhi Government General Hospital (a tertiary care teaching institution in Chennai, Tamil Nadu, India), with a 60% mortality rate despite active management, thus elucidating its rarity and severity.

## Case presentation

A 25-year-old male presented to our emergency department with complaints of diffuse headache and marked fatigue after consumption of approximately 15-20 mL of crop-flowering stimulant (20% nitrobenzene) an hour ago with an alleged suicidal intention. The patient is a chronic alcoholic with no known comorbidity.

**Figure 1:**
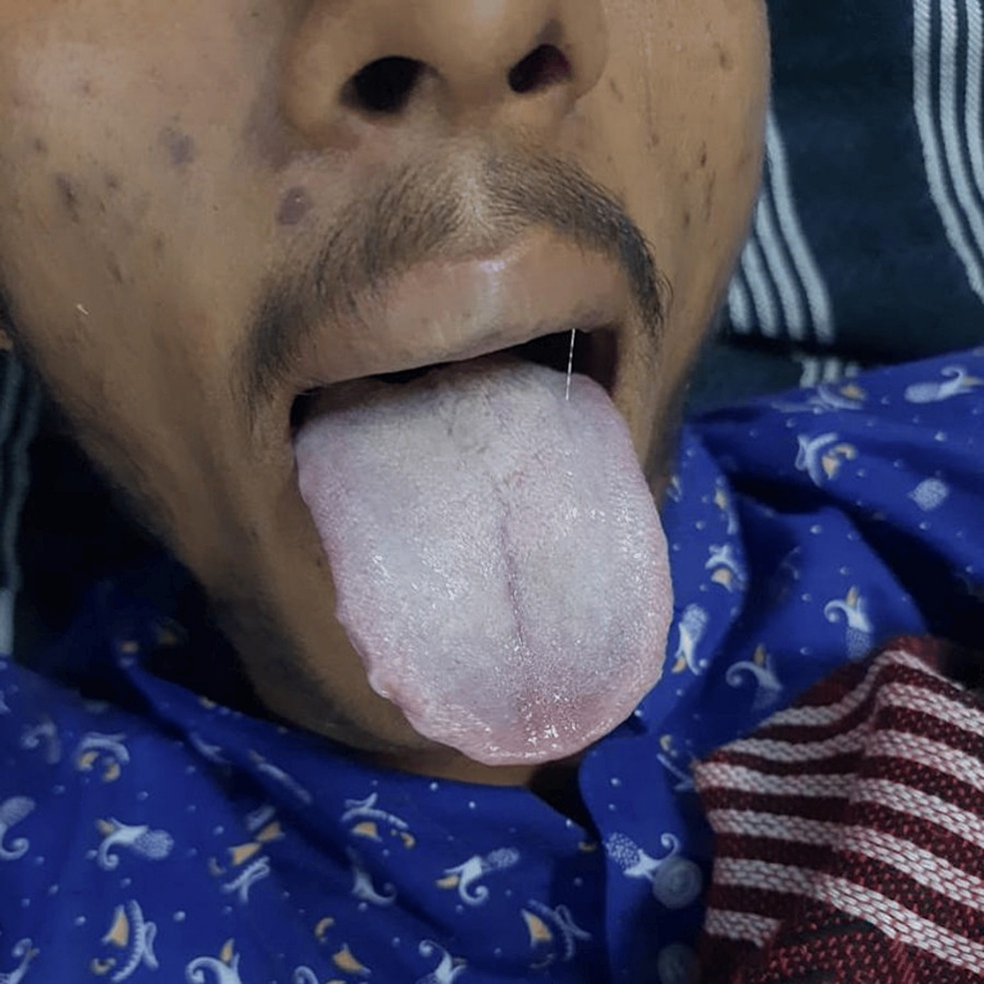
Cyanosis of the tongue after ingestion of 20% nitrobenzene

The patient was conscious, oriented, and afebrile with a Glasgow Coma Scale score of 15/15. On examination, he had central and peripheral cyanosis (Figure 1). Blood pressure was 120/80 mm of Hg, heart rate was 92 beats/minute, respiratory rate was 16 breaths/minute, and oxygen saturation (SpO_2_) was 76% on room air, which did not improve with high-flow oxygen at 15 L/minute via a non-rebreathing mask. Pupils were normal and reactive to light on both sides. On auscultation, normal vesicular breath sounds were heard and no murmurs were noted.

**Figure 2:**
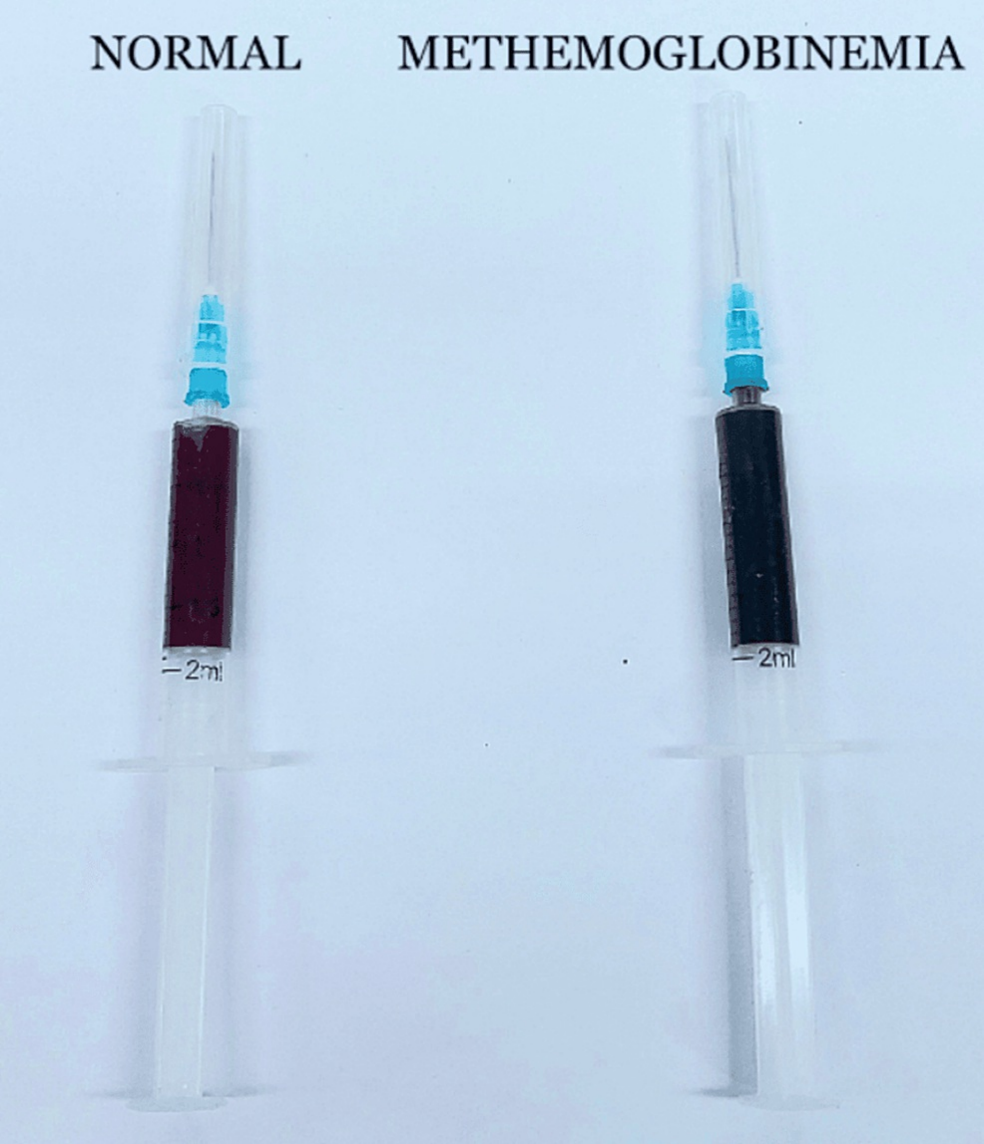
Chocolate-brownish discoloration of blood sample due to methemoglobinemia

Gastric lavage was done with sodium bicarbonate followed by activated charcoal (1 g/kg of 20% suspension), and IV fluids were started. An arterial blood sample was collected and was found to be chocolate-brown in color (Figure 2). Arterial blood gas analysis reported a pH of 7.39, partial pressure of oxygen (PaO_2_) of 86 mmHg, arterial oxygen saturation (SO_2_) of 97%, partial pressure of carbon dioxide (pCO_2_) of 39 mmHg, and bicarbonate (HCO_3_^-^) of 21 mEq/L. Blood cell counts, liver enzymes, and serum creatinine were found to be within normal limits. Chest X-ray and ECG showed a normal study. Due to the significant oxygen saturation gap of 21%, the possibility of methemoglobinemia was considered. Further investigation revealed the serum methemoglobin level to be 22% (normal: 0-2% of hemoglobin).

The patient was initiated on IV methylene blue at a dose of 1 mg/kg as a 1% solution in 100 mL normal saline over five minutes. Oral ascorbic acid 1.5 g was given immediately and thrice a day thereafter. Forced diuresis was instituted to bring down the MetHb levels in the blood. IV thiamine 100 mg was given thrice a day, owing to the patient’s history of chronic alcoholism.

The patient showed gradual clinical improvement, and on day three, cyanosis had resolved, and SpO_2_ was 96% in room air (Table 1). He was shifted to the ward and subsequently discharged with oral ascorbic acid tablets. Psychiatric counseling was given prior to discharge. On follow-up after two weeks, the patient was found to be clinically normal with no residual symptoms.

**Table 1:** Arterial blood gas analysis of the patient on admission day and day three PaO_2_: partial pressure of oxygen; SO_2_: arterial oxygen saturation; SpO_2_: oxygen saturation; pCO_2_: partial pressure of carbon dioxide; HCO_3_^-^: bicarbonate.

	On admission day	On day 3	Normal values
pH	7.39	7.41	7.35 – 7.45
PaO_2_	86 mmHg	90 mmHg	75 – 100 mmHg
SO_2_	97%	96%	95 – 100%
SpO_2_ (in room air)	76%	96%	95 – 100%
pCO_2_	39 mmHg	40 mmHg	35 – 45 mmHg
HCO_3_^-^	21 mEq/L	23 mEq/L	22 – 26 mEq/L
MetHb	22%	2%	0 – 2%

## Discussion

Nitrobenzene (also called oil of mirbane or nitrobenzol) is an aromatic compound with the chemical formula C_6_H_5_NO_2_. It is a water-insoluble yellow liquid with an almond-like odor. Most of the commercially produced nitrobenzene is hydrogenated to aniline, which is the precursor for the manufacture of azo dyes, paints, synthetic rubber, lubricating oil, explosives, pharmaceuticals, and crop-flowering stimulants. Nitrobenzene is easily absorbed through the gastrointestinal tract, respiratory route, and skin following exposure, and accumulates in the brain, liver, stomach, adipose tissue, and blood due to its lipophilic nature. The in vivo metabolism of nitrobenzene occurs by both oxidation and reduction reactions. Oxidative reactions yield nitrophenols and phenylhydroxylamine, while reduction yields nitrosobenzene, phenylhydroxylamine, and aniline (which further undergoes phase II glucuronidation reactions). The metabolites of nitrobenzene are likely associated with the toxicological effects of the compound. Intestinal microbes have been shown to mediate their formation. The major route for excretion from the body is through the urinary excretion of metabolites (mainly, p-aminophenol and p-nitrophenol). Toxicity in nitrobenzene poisoning is due to the excessive oxidation of the ferrous (Fe^2+^) moiety of hemoglobin to ferric (Fe^3+^) state, thus impairing its oxygen-carrying capacity. The resultant MetHb imparts a brownish discoloration to the blood. Two intrinsic mechanisms keep a check on the accumulation of MetHb in the blood. The first mechanism is mediated by glutathione produced from the nicotinamide adenine dinucleotide phosphate (NADPH) from the hexose monophosphate (HMP) shunt pathway in the RBCs. The other mechanisms rely on diaphorase-I (utilizes nicotinamide adenine dinucleotide hydrogen (NADH) through cytochrome b_5_ reductase) and diaphorase-II (utilizes NADPH) enzyme systems for MetHb reduction. An acute episode of intoxication overloads these mechanisms, thus resulting in MetHb toxicity [2].

The symptomatology of nitrobenzene poisoning depends on the concentration of MetHb in the blood. The normal blood level of MetHb is between 0% and 2%. Patients are asymptomatic for up to 10-15% of intoxication and may present only with cyanosis. Symptoms like chest pain, headache, tachycardia, and tachypnea occur when the MetHb levels rise above 20%. The clinical picture is complicated by lethargy, disorientation, and metabolic acidosis at 40-50% levels, leading to ventricular arrhythmias, elevated blood pressure, seizures, and coma. The lethal dose of nitrobenzene ranges between 1 and 10 g, which produces fatal MetHb concentrations of about 70%. Patients may present with hepatosplenomegaly, elevated liver enzymes, leukocytosis, and hemolytic anemia. The clinical features of intoxication can persist for days (or recur after a few days) despite adequate management due to the slow turnover and release of nitrobenzene from its tissue stores into the blood over time. Since the symptoms depend on the proportion of MetHb in the blood, patients with anemia or glucose-6-phosphate dehydrogenase enzyme deficiency suffer from symptoms on the severe end of the spectrum [3].

Suspicion of nitrobenzene poisoning should be raised with a history of poison ingestion with the smell of bitter almonds, central and/or peripheral cyanosis that persists despite oxygen therapy in the absence of other etiologies, and a significant oxygen saturation gap (difference between SO_2_ on arterial blood gas analysis and SpO_2_ from pulse oximeter) of greater than 5%. The chocolate brown color of the blood sample, which does not turn bright red on agitation, is due to MetHb accumulation. This can be taken as a diagnostic clue and is the basis for the bedside test for methemoglobinemia [4].

Confirmatory tests include spectroscopic methods like co-oximetry and urinary presence of nitrobenzene metabolites like p-nitrophenol and p-aminophenol. It is also important to rule out other causes of methemoglobinemia (congenital or acquired) before a definitive diagnosis of nitrobenzene poisoning can be made. Sulfhemoglobinemia is a close differential diagnosis [5]. MetHb estimation has great utility in making management decisions and assessing patient prognosis following treatment.

Management of nitrobenzene poisoning is on the lines of decontamination and supportive management. The goal is to remove the unabsorbed poison or excrete the absorbed fraction from the body and to attain maximum reduction of MetHb back to normal hemoglobin to regain its oxygen-carrying potential. Methylene blue is the antidote of choice for acquired methemoglobinemia. It works by utilizing the NADPH from the HMP shunt pathway and gets converted into leucomethylene blue, which reduces MetHb to hemoglobin. It is administered IV as a 1% solution in normal saline at a dose of 1-2 mg/kg over five minutes and may be repeated after an hour if required. Since NADPH production through the HMP shunt pathway requires glucose, dextrose can be given to improve the effectiveness of the therapy. Repeated low doses of methylene blue may be considered if fluctuating symptoms occur due to the release of the toxin from tissue stores. Oral preparations have also been found to be effective (bioavailability of 72%) at a dose of 2 mg/kg and can be used in resource-limited settings [6]. The use of methylene blue is contraindicated in G6PD deficiency due to the risk of fatal hemolysis [7]. Passage of blue-green urine is normal during the course of therapy, which occurs as the unchanged blue pigment combines with urochrome in the urine.

Ascorbic acid is an antioxidant and free radical scavenger, which reduces nicotinamide adenine dinucleotide (NAD^+^) to NADH. It is given to patients with MetHb levels greater than 30%, in those with contraindications for methylene blue (high dose 10 g IV given), or simply as an adjuvant or maintenance measure. It is found to be effective at a dose of 0.5-1 g IV thrice a day in 5% dextrose solution, or at an oral dose of 1.5 g thrice a day. Recent studies have shown that N-acetyl cysteine (NAC) can also attain a reduction of MetHb [3]. Several mechanisms have been proposed for the action of NAC, including restoration of intracellular glutathione, direct reducing action through the sulfhydryl group on NAC, and anti-oxidant action by reducing local inflammation.

Decontamination and supportive measures must be pursued simultaneously during the management. Gastric lavage with activated charcoal and forced emesis with polyethylene glycol are effective decontamination modalities. Forced diuresis can bring down the blood MetHb levels drastically. The patient must be maintained on high-flow oxygen, and vasopressors can be used for maintaining tissue perfusion. Metabolic acidosis can be countered by administering sodium bicarbonate intravenously or intermittent hemodialysis may be considered. Adequate urine output should be maintained, and patients should be advised about proper nutrition. RBC exchange transfusion and hyperbaric oxygen therapy are reserved for patients not responding to the conventional modalities of management [3].

Since the event of poisoning in our patient had occurred with suicidal ideation, psychiatric assessment and counseling were offered prior to discharge, and the patient was encouraged for regular follow-up sessions. Chronic alcoholics must be offered intravenous thiamine 100 mg thrice a day and oral thiamine after discharge, considering the possibility of Korsakoff psychosis as an etiology for the suicidal intent.

## Conclusions

Nitrobenzene poisoning is a rare yet life-threatening challenge. The resultant acute methemoglobinemia has a high mortality rate, thus early aggressive management must be pursued. Even consumption of small amounts of nitrobenzene can produce high levels of methemoglobinemia within a short span, as seen in our patient. Early detection of poisoning can be done based on the bitter almond odor of the poison, brownish discoloration of blood, and significant saturation gap not responding to oxygen therapy.

Methylene blue and ascorbic acid are the mainstay for management. They must be promptly administered to the suspected patients without delay. Decontamination measures must be undertaken as deemed fit for the patient. Oxygen therapy and correction of metabolic acidosis must be done for all patients. Thus, a high degree of suspicion and early identification of methemoglobinemia with timely administration of methylene blue prevents the various detrimental effects of the poisoning and ensures a better prognosis for the patients.
